# A Logical Framework (MYGERYFS) for Hospital Foodservice to Prevent Malnutrition Among Geriatric Patients in Hospitals, Malaysia: Protocol for a Feasibility Study

**DOI:** 10.2196/42496

**Published:** 2023-01-31

**Authors:** Noraida Omar, Shazli Illyani Shafiee, Siti Hazimah Nor'hisham, Zuriati Ibrahim, Rosita Jamaluddin, Syafiqah Rahamat, Barakatun Nisak Mohd Yusof, Halimatus Sakdiah Minhat, Hakimah Sallehuddin, Nur Syazwani Mazlan

**Affiliations:** 1 Department of Dietetics Faculty of Medicine and Health Sciences Universiti Putra Malaysia Selangor Malaysia; 2 Malaysian Research Institute on Ageing (MyAgeing™) Universiti Putra Malaysia Selangor Malaysia; 3 Department of Community Health Faculty of Medicine and Health Sciences Universiti Putra Malaysia Serdang, Selangor Malaysia; 4 Geriatric Unit, Department of Medicine Faculty of Medicine and Health Sciences Universiti Putra Malaysia Serdang, Selangor Malaysia; 5 Department of Economics School of Business and Economics Universiti Putra Malaysia Serdang, Selangor Malaysia

**Keywords:** hospital foodservice satisfaction, hospital meals satisfaction, elderly, nutrition, malnutrition in elderly, malnutrition, geriatric, patient, prevention, nutrient, feasibility

## Abstract

**Background:**

Geriatric malnutrition in hospitals is common and can be affected by many things, including poor satisfaction toward hospital foodservice. Hospital foodservice plays an important role in a patient’s recovery process by providing adequate nutrients. On top of that, patients’ foodservice satisfaction can easily be afflicted by the quality of food served and the overall foodservice experience. Furthermore, malnutrition can occur from poor foodservice quality, especially among geriatric patients.

**Objective:**

This study aims to assess the effectiveness of the Malaysian Geriatric Patients’ Hospital Foodservice Protocol (MYGERYFS).

**Methods:**

The protocol comprises 3 phases. Phase One is a cross-sectional study that took place at public hospitals with geriatric wards in the Klang Valley. Univariate data from Phase One were analyzed descriptively. Pearson correlation and chi-square were conducted to find factors associated with foodservice satisfaction. Phase Two involves the collaboration of health care professionals in the geriatric field. In Phase Three, a feasibility study will be conducted to determine the feasibility of the MYGERYFS protocol in a hospital among 60 geriatric patients. These patients will be randomized into control and intervention groups, respectively. Intervention care will be done to ensure the safety of the protocol.

**Results:**

Data collection for Phase One of the study has been completed. A total of 233 geriatric respondents with the mean age of 71.39 (SD 7.99) years were gathered. Approximately 51.5% (n=120) of the respondents were female, while 48.5% (n=113) were male, with a mean BMI of 24.84 (SD 6.05) kg/m2. Their mean energy and protein intakes were 1006.20 kcal (SD 462.03 kcal) and 42.60 (SD 22.20) grams, respectively. Based on the Mini Nutritional Assessment, older patients who scored 12-14 (normal) were 27.9% (n=65), those who scored 8-11 (at risk) were 54.9% (n=128), and those who scored 0-7, which is the lowest (malnutrition), were 17.2% (n=40) of the study population. Hence, most patients were at risk of malnutrition. Although a majority of the patients claimed to have good foodservice satisfaction 26.2% (n=61), they also experienced at least 3 barriers during mealtimes. It was found that dietary intake and mealtime barriers were significantly associated with the respondent’s foodservice satisfaction. Data for Phase Two and Phase Three are yet to be collected and analyzed.

**Conclusions:**

This study protocol could potentially benefit the hospital foodservice system and aid in improving geriatric nutritional status.

**Trial Registration:**

ClinicalTrials.gov NCT04858165; https://clinicaltrials.gov/ct2/show/NCT04858165

**International Registered Report Identifier (IRRID):**

RR1-10.2196/42496

## Introduction

“Older people” is defined as individuals aged 65 years or older who are deemed suitable to receive the national pension plan [1]. According to Orimo et al [1], the range of older persons can be divided into two categories. They are (1) “early elderly” (individuals aged 65-74 years) and (2) “late elderly” (individuals aged ≥75 years). In Malaysia, “elderly” is recognized as those aged 60 years and older, due to its policy and program development purposes [2]. The increasing trend of the aging population has shifted the world’s demographic patterns [3]. As a developing country, Malaysia is not spared from this phenomenon [4]. The recent National Health Morbidity Survey (2018) estimated that the country’s older population is at 2.3 million (6.3% of the total population) and is predicted to double within 23 years [2]. The aging process often relates to health complications [2]. Apart from the presence of comorbidities, malnutrition in older people is also common [5].

The reasons for having compromised nutritional status among older individuals are changes in physical, cognitive, psychological, and social factors (eg, living alone), as well as having limited income [6]. Other risk factors include the presence of comorbidities [7,8], poor dental status [9], and inadequate dietary intake [7,10,11]; moreover, those living in health care institutions often complain of having poor foodservice experience [12,13]. Older people with malnutrition often find themselves in a state of dependency, having prolonged hospital stays, an increase in infection rate that eventually diminishes their quality of life, and an increase in health care costs and mortality [6,7,14].

There are countless studies investigating malnutrition among hospitalized older individuals, and the prevalence is indisputable. The global prevalence of malnutrition in hospitalized older patients ranges from 12% to 75% [7]. Consistent with the worldwide trend, Malaysia also shows a prevalence of malnutrition that ranges from 21% to 55% among hospitalized geriatric patients, which appears to be a matter of concern [15].

Despite the interventions carried out thus far, the prevalence of malnutrition among geriatric patients is still appalling [16]. Most older people view themselves as healthy, in spite of their sociodemographic status (educational level, income status, and daily living activities) [17]. Despite their healthy self-image, older individuals are still vulnerable to malnutrition due to their frail condition [6]. In addition, of 52% of malnourished older patients, 18% were found to be nutritionally compromised even before admission [18]. Surprisingly, these older adults even believe that having inadequate nutritional intake is normal without realizing the consequences that malnutrition carries [6].

Hospital meal is considered as one of the important components of care. Hospital meals are served not only to facilitate well-being and recovery but also to contribute to the patients’ satisfactions on their overall treatment experiences [19]. Looking at the hospital foodservice system, oftentimes the patients’ nutritional status is impacted by it. For example, hospital-acquired malnutrition is believed to be caused by the poor quality and appearance of the food and its environment [20]. The same study reported that food quality is found to be the best predictor of patients’ overall satisfaction, followed by service quality given by staff. A study in Australia has proven this by introducing a room service model to hospitalized patients. The results showed good upturns in the patients’ nutritional intake and satisfaction as well as reduced plate waste and patient meal costs after the method was implemented [21].

Another study reported that patients found it tiring to order and eat their food at specific times [22]. On the contrary, these patients were also sensitive to delays and poor service. Further, the study documented that patients appreciated the presence of nonmedical foodservice staff, but at the same time felt pressured by them. According to a study conducted in a military hospital in Turkey, the patients’ satisfaction levels on the hospital’s foodservice system showed inconsistency. The results showed that 51.3% evaluated the foodservice quality to be adequate, while 32.4% deemed it was of inadequate quality. Accordingly, the remaining 16.3% stated that they were uncertain [23]. The different spectrum of thoughts by the patients only makes it hard to fathom, thus affecting the ways of intervention in many hospitals’ foodservice systems. Therefore, the redesigning of hospital foodservice models may be needed not only to drive toward the improvement of patient satisfaction and cost but also to influence clinical outcomes associated with nutritional intake.

**Figure 1:**
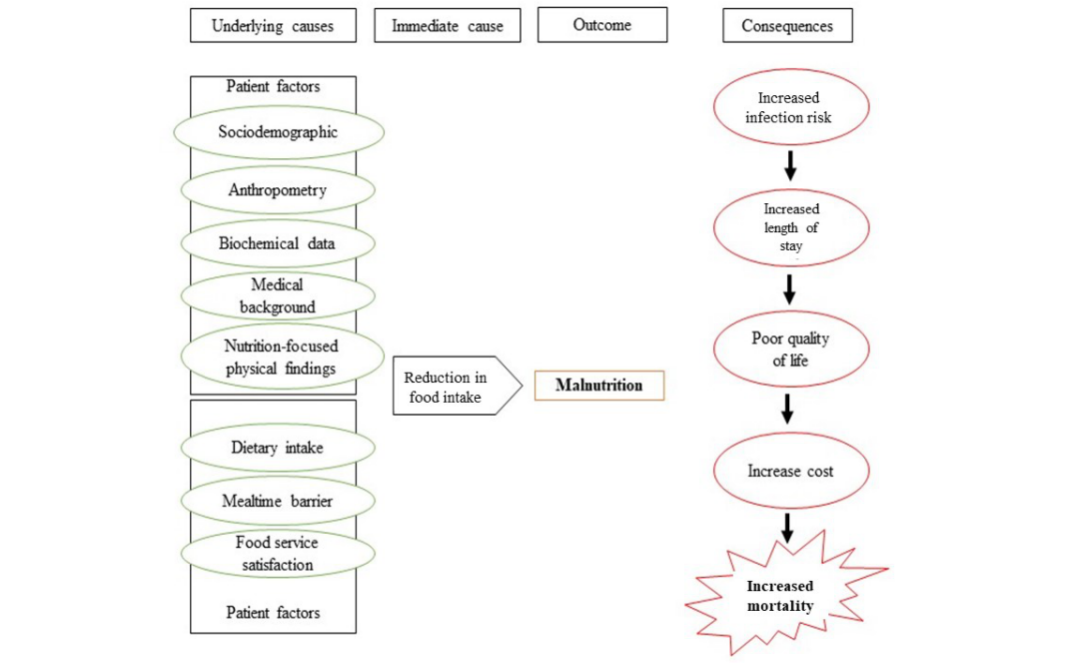
Conceptual framework of the causes of malnutrition and its consequences.

Figure 1 shows the conceptual framework of the causes of malnutrition and its possible consequences. This study aims to develop, implement, and evaluate the effectiveness of a Malaysian Geriatric Patients’ Hospital Foodservice Protocol (MYGERYFS) in order to improve both the nutritional status of hospitalized geriatric patients and the quality of the foodservice.

## Methods

### Study Design

The MYGERYFS project is a 2 year and 6 months intervention program, conducted among malnourished older individuals with pre- and postintervention to assess the effectiveness of the protocol. The trial comprises the nutritional status of hospitalized geriatric patients aged 60 years and above in 5 public hospitals in the Klang Valley, which are as follows: (1) Hospital Selayang, (2) Hospital Sungai Buloh, (3) Hospital Kuala Lumpur, (4) Hospital Tengku Ampuan Rahimah, and (5) Hospital Pengajar Universiti Putra Malaysia.

The study comprises the following phases: (1) Phase One—a preliminary study that identified the prevalence and factors associated with hospitalized geriatric malnutrition; (2) Phase Two—protocol development with health care professionals; and (3) Phase Three—testing the feasibility of the protocol. The trial has been registered at ClinicalTrials.gov (NCT04858165) on March 30, 2021.

### Phase One

The first phase of the MYGERYFS is a cross-sectional study conducted to assess the recent nutritional status of older hospitalized patients and the factors associated. Phase One has been completed and analyzed in August 2022. Using the proportion formula by Cochran (1963) [19], a total of 233 geriatric patients were gathered as samples for this study.

In this phase, the patients or their respective caregivers were interviewed by the researchers. A series of questionnaires that hold information on the patients’ sociodemographic and medical background, anthropometry measurements, dietary intake, mealtime barriers, foodservice satisfaction levels, and nutritional statuses were put together. The collected data were assessed and labeled as preliminary data.

### Phase Two

Phase Two of the MYGERYFS protocol studies the current feeding practices and opinions of health care professionals. A group of geriatric health experts, specifically geriatricians, nurses, and dietitians, will be invited to review and discuss the preliminary data. Subsequently, the existing clinical procedures will be audited and improved in line with the recent research. The protocol will be finalized in a consensus meeting.

### Phase Three

The newly developed protocol will be tested in a single institution. Altogether, 60 geriatric patients will be recruited. These patients will be divided into 2 groups (control and intervention groups). The control group patients will be given usual hospital care, and the intervention group patients will be cared for according to the developed protocol for approximately 3 consecutive days. Dietary recall of 3 days was thought to be better than 24-hour recall or 7-day recall [24]. This is because a single 24-hour recall could lead to overestimation of intake, while a dietary recall of more than 3 days often leads to lower intake as respondents feel burdened to report their intake further, thus omitting their food items altogether [24]. Details on their dietary adequacy and clinical outcomes will be documented. The feasibility and safety of the protocol will be evaluated. The findings will provide fundamental data to develop a foodservice protocol for Malaysian geriatric patients.

### Participants

#### Ethics Approval

This study was approved by the National Medical Research Register for Phase One (NMRR-20-308-52632) and was reapplied for Phase Three. In Phase One, a written consent form will be sought from all respondents before the study. Permission from each of the hospitals’ clinical research centers was obtained before data collection.

#### Screening of Nutritional Status

Five public hospitals in the Klang Valley were listed and approached for Phase One of the study. These hospitals were purposely selected due to the availability of their geriatric wards. The selected patients will be screened using the Mini Nutritional Assessment Short Form (MNA-SF). The MNA-SF evaluates 5 components of common geriatric problems, which are food intake, weight status, mobility status, presence of psychological problems, and neurological stress level. These components will be scored based on the patients’ conditions and are categorized into the following: (1) normal nutritional status (12-14 points), (2) at risk of malnutrition (8-11 points), and (3) malnourished (0-7 points) [25].

#### Eligibility and Recruitment

Both Phase One and Phase Three of this study shared similar inclusion and exclusion criteria, as mentioned in Textbox 1.

**The summary of the inclusion and exclusion criteria for Phase One and Phase Three of the Malaysian Geriatric Patients’ Hospital Foodservice Protocol (MYGERYFS).**

**Inclusion criteria**

- Patients aged 60 years and older
- Able to understand and speak Malay or English or both
- Patients who are on an oral diet or Oral Nutrition Support or both
- Patients who are admitted for more than 48 hours

**Exclusion criteria**

- Patients with mild dementia or adjustment disorders that are common in geriatric patients
- Critically ill patients
- Patients who are on full enteral or parenteral feeding

#### Sample Size Calculation

The sample size for Phase One of the study will be calculated using the proportion formula by Cochran (1963) [19]. A total of 233 geriatric patients will be gathered as samples for this study.

Meanwhile, Phase Two will only require the cooperation of health care professionals; thus, no sample size calculation will be calculated.

In Phase Three of the study, 60 geriatric patients will be recruited and divided equally into control and intervention groups. Therefore, a sample of 30 respondents per arm will be required. As suggested by Cohen [25], a sample size of 30 in each arm is needed to detect the differences between groups, which should lead to about a minimum of 80% power in a study.

#### Randomization and Allocation

Patients in Phase Three of the study will be randomized into control and intervention groups, respectively. To do so, patients will be allocated with the help of a computer-generated random number sequence.

### Intervention

Preceding the development of the MYGERYFS protocol, a feasibility study will be conducted during Phase Three. The study will be carried out in a single institution with a total of 60 geriatric patients. These patients will be screened for their eligibility and separated into control and intervention groups, respectively. Those in the control group will receive the usual hospital care, while the other group will experience hospital care according to the developed protocol. Both groups will be observed for their dietary intake for approximately 2 days from the day of the intervention. All of this information will be compared within the 2 groups.

### Outcome Measures

#### Primary Outcome

##### Nutritional Status

The nutritional statuses of geriatric patients were assessed using the MNA-SF. The MNA-SF evaluates 5 components of common geriatric problems, which are food intake, weight status, mobility status, presence of psychological problems, and neurological stress level. These components were scored based on the patients’ conditions and categorized into the following: (1) normal nutritional status (12-14 points), (2) at risk of malnutrition (8-11 points), and (3) malnourished (0-7 points) [25].

##### Foodservice Satisfaction

The patients’ levels of foodservice satisfaction were documented by the validated Acute Care Hospital Foodservice Patient Satisfaction questionnaire by Capra [26] in 2005 via an interviewing method. The questionnaire weighs 4 main foodservice aspects, which are food quality, meal service quality, staff or service issues, and physical environment. The higher the mean score is, the better the results will be [26].

##### Dietary Intake

The amount of food consumed by the respondents in 2 days was recorded and analyzed using Nutritionist Pro (version 2.4.1, First Data Bank) [27]. From these data, the patients’ energy and protein adequacy was compared against their requirements.

#### Secondary Outcomes

##### Anthropometric Measurements

The anthropometric measures considered for this study are as follows:

- Body weight was measured using an electronic scale (digital weighing scale; OMRON HBF-357) according to the standard techniques [28].
- Estimation of weight is done for patients who were unable to stand without assistance using knee height measurement and mid-upper arm circumference. Weight was measured using equations developed by Ross Laboratories [29].
- Height was measured using a stadiometer (SECA height rod) according to the standard techniques [30].
- Estimation of height is done for patients with distinctive conditions (eg, kyphosis, scoliosis) using knee height measurement. Height was calculated using equations developed for older Malaysian people [31].
- BMI was calculated using the following equation: weight in kilograms divided by height in meters squared—weight (kg)/height^2^(m^2^) [30].

##### Mealtime Barriers

The patients’ mealtime barriers were determined and scored using the Mealtime Audit Tool questionnaire. This questionnaire prioritizes the types of potential barriers during mealtime. Each question is marked with a “Yes” or “No” answer. Scoring for this questionnaire is based on the total “No” responses. This value represents the number of barriers faced by the patients. The higher the “No” responses were, the more barriers were experienced [32].

### Statistical Analysis

Data were entered and analyzed using SPSS 25.0 (IBM Corp). In Phase One of the study, descriptive statistics of the continuous data were presented using mean and standard deviation. Meanwhile, categorical data were presented as frequencies and percentages. To analyze the associations of malnutrition, Pearson coefficient correlation and chi-squared test were used. Statistical significance was set at *P*<.05.

In Phase Two, no analysis will be conducted as it only requires the discussion of health care professionals.

In Phase Three, the descriptive statistics will be presented using mean and standard deviation for continuous data. On the other hand, categorical data will be presented as frequencies and percentages. To compare the control and intervention groups, an independent 2-tailed *t* test will be used. Statistical significance is set at *P*<.05.

## Results

Phase One of the protocol has already started. A total of 233 older respondents participated in this study. The mean age was 71.39 (SD 7.99) years, where most 51.5% (n=120) of the respondents were female and the rest 48.5% (n=113) were male.

The mean BMI of the older respondents was 24.84 (SD 6.05) kg/m^2^. Based on the results showed, most (n=110, 47.2%) participants were classified as underweight, 37.8% (n=88) were normal, and 17.2% (n=40) were either overweight or obese.

The mean energy and protein intakes were 1026.82 kcal (SD 457.66 kcal) and 43.54 (SD 22.66) grams, respectively. The majority of the respondents 70.8% (n=165) had inadequate energy intake (<25-30 kcal/kg). The same trend was shown for their protein intake. Approximately 77.7% (n=181) of them consumed protein below 1.2 g/kg. It was also found that 84.5% (n=197) did not receive oral nutrition support in ward.

The percentage of older patients in hospitals in the Klang Valley who responded to the MNA-SF questionnaire—which was divided into 3 scores of “Normal” (12-14), “At Risk” (8-11), and “Malnutrition” (0-7)—was 27.9% (n=65) normal, 54.9% (n=128) at risk, and 17% (n=40) malnutrition, which is the lowest. Thus, most patients were at risk of malnutrition.

The mean Mealtime Audit Tool score was 3.27 (SD 1.89), showing that the respondents experienced at least 3 barriers during mealtimes. The most common barriers that these patients came across were as follows: (1) respondents were not paid any visits by health care staff midmeal to see if they need anything, (2) respondents were not asked or given other food if they refused to eat their meal, and (3) respondents were not offered to go to the washroom before mealtime. Most of the respondents 74.7% (n=119) experienced good foodservice satisfaction while the other 25.4% (n=59) did not.

Pearson correlation test was done to find the associations between BMI, dietary intake, mealtime barriers, and nutritional status with foodservice satisfaction. According to Table 1, there was no association found between BMI and foodservice satisfaction among hospitalized geriatric patients (*r*=–0.056, *P*=.39). As for the dietary intake, both energy (*r*=0.337, *P*<.001) and protein (*r*=0.255, *P*<.001) were significantly associated with foodservice satisfaction. A statistically significant result was also shown between mealtime barriers and foodservice satisfaction (*r*=–0.225, *P*<.01).

A chi-square test was also conducted between nutritional status with foodservice satisfaction. No significant association was found between malnutrition with foodservice satisfaction (*χ^2^_8_*=12.778, *P*=.12).

**Table 1:** Pearson correlation between BMI, dietary intake, and mealtime barriers with foodservice satisfaction.

Variables	Foodservice satisfaction statistics	
	*r*	*P* value
BMI	–0.056	.39
Average energy intake	0.337	<.001^a^
Average protein intake	0.255	<.001^a^
Mealtime barriers	–0.225	.07^a^

^a^Correlation is significant at *P*<.001.

## Discussion

### Principal Findings

Based on the results, it can be seen that the patients’ dietary intake was associated with their foodservice satisfaction. Improvement on protein and energy intake to some extent had improved patients’ satisfaction within a short period of hospital stay [26].

Next, having mealtime barriers can also affect the overall foodservice satisfaction. This results negatively in the overall experience of services, including foodservice, even though the differences were not statistically significant [33].

This study has its limitations. Phase One of the study was conducted cross-sectionally, which constrained us from finding its causality. Moreover, the feasibility study that will be done may also have its negative impact. This is because feasibility studies often do not identify new concepts as they are based on assumptions. Thus, clear findings must be analyzed during Phase One, and clear decisions need to be made in Phase Two in order to prove them to be realistic.

For all we know, endless research on geriatric malnutrition has been conducted, and the results remain significantly high. To understand the issue, various factors will be studied and addressed with geriatric malnutrition. Thus, the development of the MYGERYFS is considered an unprecedented approach toward delivering a better health care quality for geriatric patients in hospitals, with an ultimate aim to improve the nutritional status of geriatric patients as well as to reduce further deterioration of nutrient intake among this population.

Besides, with the existence of this protocol, there will be a chance to further enhance public hospitals’ foodservice system. The provision of a variety of nutritious meals for these patients is believed to be useful with the protocol as an aide. Each meal served to the patient will be tailored to their specific health conditions.

Another strength of this protocol is the encouragement of providing mealtime assistance to those in need. This modified service may enhance the patients’ overall foodservice satisfaction. A collaboration between the health care and the foodservice system to improve the well-being of geriatric patients is imaginable.

### Conclusions

In sum, the MYGERYFS protocol is designed to suit the needs of geriatric patients in hospitals. With the help of other health care professionals, we assure that this protocol will be able to minimize geriatric malnutrition and enhance the patients’ overall foodservice satisfaction. Should this protocol show positive effects, the strategies included may be of help to other Malaysian older populations as well.

## Abbreviations

MNA-SF: Mini Nutritional Assessment Short Form
MYGERYFS: Malaysian Geriatric Patients’ Hospital Protocol
